# The use of the mannitol test as an outcome measure in asthma intervention studies: a review and practical recommendations

**DOI:** 10.1186/s12931-021-01876-9

**Published:** 2021-11-07

**Authors:** Asger Sverrild, Joanna Leadbetter, Celeste Porsbjerg

**Affiliations:** 1 Department of Respiratory Medicine, Bispebjerg Hospital, Copenhagen University, Ebba Lunds vej 48, 2400 Copenhagen, Denmark; 2 Statistics Manager, Pharmaxis, Frenchs Forest, NSW Australia

**Keywords:** Airway hyperresponsiveness, Asthma, Bronchoprovocation, Outcome measure, Intervention studies, PD_15_, Mannitol

## Abstract

**Background:**

The mannitol test is an indirect bronchial challenge test widely used in diagnosing asthma. Response to the mannitol test correlates with the level of eosinophilic and mast cell airway inflammation, and a positive mannitol test is highly predictive of a response to anti-inflammatory treatment with inhaled corticosteroids. The response to mannitol is a physiological biomarker that may, therefore, be used to assess the response to other anti-inflammatory treatments and may be of particular interest in early phase studies that require surrogate markers to predict a clinical response. The main objectives of this review were to assess the practical aspects of using mannitol as an endpoint in clinical trials and provide the clinical researcher and respiratory physician with recommendations when designing early clinical trials.

**Methods:**

The aim of this review was to summarise previous uses of the mannitol test as an outcome measure in clinical intervention studies. The PubMed database was searched using a combination of MeSH and keywords. Eligible studies included intervention or repeatability studies using the standard mannitol test, at multiple timepoints, reporting the use of PD_15_ as a measure, and published in English.

**Results:**

Of the 193 papers identified, 12 studies met the inclusion criteria and data from these are discussed in detail. Data on the mode of action, correlation with airway inflammation, its diagnostic properties, and repeatability have been summarised, and suggestions for the reporting of test results provided. Worked examples of power calculations for dimensioning study populations are presented for different types of study designs. Finally, interpretation and reporting of the change in the response to the mannitol test are discussed.

**Conclusions:**

The mechanistic and practical features of the mannitol test make it a useful marker of disease, not only in clinical diagnoses, but also as an outcome measure in intervention trials. Measuring airway hyperresponsiveness to mannitol provides a novel and reproducible test for assessing efficacy in intervention trials, and importantly, utilises a test that links directly to underlying drivers of disease.

**Supplementary Information:**

The online version contains supplementary material available at 10.1186/s12931-021-01876-9.

## Introduction

Asthma is heterogeneous in its manifestations and response to treatment [1]. The development of novel asthma treatments can be both time consuming and costly, making the demonstration of clinical efficacy early in a treatment’s development process essential [2].

### Demonstrating clinical efficacy

The methods used to assess the efficacy of asthma treatments currently focus on outcomes such as exacerbations and symptoms. Although clinically relevant, these are non-specific in terms of reflecting the underlying drivers of disease such as inflammation and airway hyperresponsiveness (AHR). Using only clinical outcome measures to assess the efficacy of a novel asthma treatment risks missing potentially relevant treatment effects—especially if tested in the wrong study population. Lung function, for example, is one of the most commonly used objective outcome measures as it is often a regulatory requirement for registration of respiratory products, but its usefulness is limited. The lung function of many patients may fall within the normal range during a consultation, and thus they are less likely to have a significant increase in lung function in response to an intervention [3].

Proof-of-concept studies are a ‘critical step’ in the early stages of clinical drug development. They provide important early evidence in a small group of patients and reflect the likelihood of a drug demonstrating clinical efficacy in subsequent larger studies. Ideally, an outcome measure for a proof-of-concept study must be standardised, repeatable, easy to perform, reflect the underlying endotype of the disease, and most importantly, must reflect any changes in clinical control after the intervention.

### Clinical efficacy—what the guidelines say

The European Medicines Agency (EMA) guideline on the clinical investigation of medicinal products for the treatment of asthma states that bronchoprotection (i.e., the ability of a drug to provide protection against bronchial challenge) is an acceptable objective measure of clinical efficacy [4]. Bronchoprotection can be assessed through direct provocation, with for example, methacholine, histamine, acetylcholine, or through indirect provocation with mannitol, adenosine monophosphate (AMP) or allergen challenge, and relates to the mechanism by which a stimulus mediates bronchoconstriction. Most current guidelines (including the EMA guideline) do not distinguish between direct and indirect measures of AHR. However, it does appear that indirect measures can be significantly more sensitive at monitoring treatment effects compared to direct measures of AHR [5, 6].

### Airway hyperresponsiveness

AHR measurement is currently used as a tool in the diagnostic work-up of potential asthmatics. Its diagnostic properties are well described and have been tested in a variety of settings ranging from unselected, population-based cohorts to occupational asthma, elite sports, military and armed forces screening, and secondary and tertiary outpatient clinics [7]. The test is generally considered highly specific (specificity around 90% depending on setting) with moderate sensitivity (around 40% to 70% in the general population) [7, 8]. Studies in patients with asthma have shown AHR to mannitol correlates with the degree of airway inflammation (eosinophils in sputum, blood and lung tissue) [9, 10], but AHR is also common in non-eosinophilic patients with asthma [11].

### The mannitol challenge test

The mannitol test was developed as an easy-to-use, standardised bronchial challenge test for diagnosing asthma in a range of clinical settings. During the test, increasing doses of dry powder preparation of mannitol is administered using an inhaler device (a nebuliser is not needed). The required dose is divided into multiple capsules which are loaded and inhaled individually [12].

**Fig. 1:**
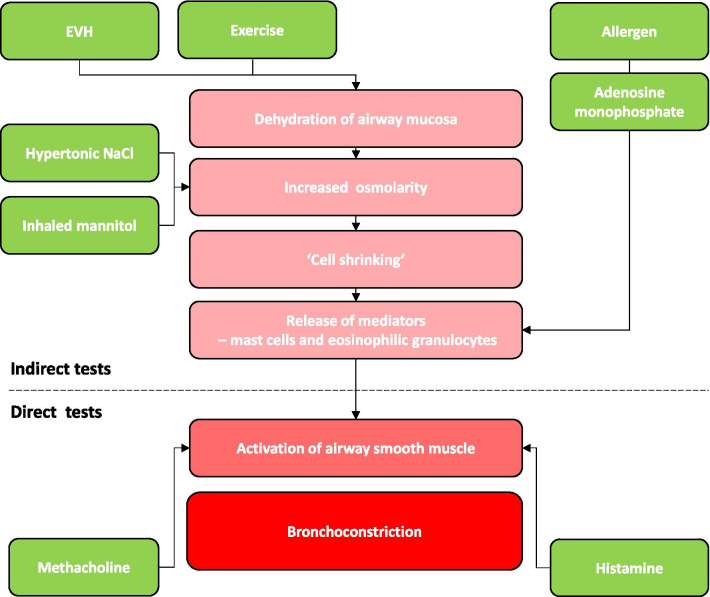
Indirect and direct bronchial provocation tests in asthma—mechanisms of action. Inhaled mannitol causes increased osmolarity of the periciliary liquid, which induces cell shrinkage as the water moves out of the cells to restore osmotic equilibrium. Through a calcium-dependent process, this leads to a release of bronchoconstricting mediators (e.g., histamine, prostaglandins and leukotrienes) from inflammatory cells, eventually causing smooth muscle contraction in responsive individuals. For further details, several reviews on the properties, safety and mode of action have been previously published [13, 14]. EVH: eucapnic voluntary hyperventilation test

The mannitol challenge test belongs to the group of indirect bronchial provocation tests (BPTs). Inhaled mannitol is thought to act through the creation of an increased osmolarity in the periciliary liquid, leading inflammatory cells (such as mast cells) to release inflammatory mediators, which eventually causes bronchoconstriction (Fig. 1). This contrasts with direct BPTs (using stimuli such as methacholine or histamine) that act directly through receptors on the airway smooth muscle to cause bronchoconstriction. It is this mechanistic difference that allows indirect provocation agents to be used to assess changes in asthma control post-intervention.

Mannitol powder for inhalation is a well-tolerated compound that is commercially available around the world and approved for use by both the EMA and the US Food and Drug Administration (FDA) as a challenge test assessing AHR. In contrast to most other AHR tests, inhaled mannitol is delivered using a small breath-actuated dry powder inhaler, has only one standard protocol, and no requirements for a nebuliser and associated calibration, or additional equipment besides a spirometer [13].

### The mannitol challenge test protocol

The challenge test kit consists of nine steps with increasing doses of mannitol (with a maximum cumulative dose of 635 mg). The patient starts by inhaling the contents of an empty (0 mg) capsule to establish baseline values (this is equivalent to the diluent step in a nebulised challenge). Forced expiratory volume in the first second (FEV_1_) is measured in duplicate (the highest measure of two reproducible manoeuvres is used). Increasing doses of mannitol (5, 10, 20, 40, 2 × 40, 4 × 40, 4 × 40, 4 × 40 mg) are administered until a decrease of at least 15% from baseline FEV_1_, a 10% decrease in FEV_1_ between two consecutive doses, or the cumulative dose reaches a total of 635 mg. The average time to a positive test result takes approximately 17 min depending on the individual’s level of AHR, and around 26 min for a negative test result [15]. As well as providing a negative or positive result, it is also a dose–response test. The provoking dose (PD) that results in a 15% drop in FEV_1_ is known as the PD_15_ of the test and is calculated based on linear interpolation on the log scale between the last and the second-last cumulative dose of inhaled mannitol and the corresponding falls in FEV_1_. This PD_15_ outcome measure will be the focus of this paper.

**Fig. 2:**
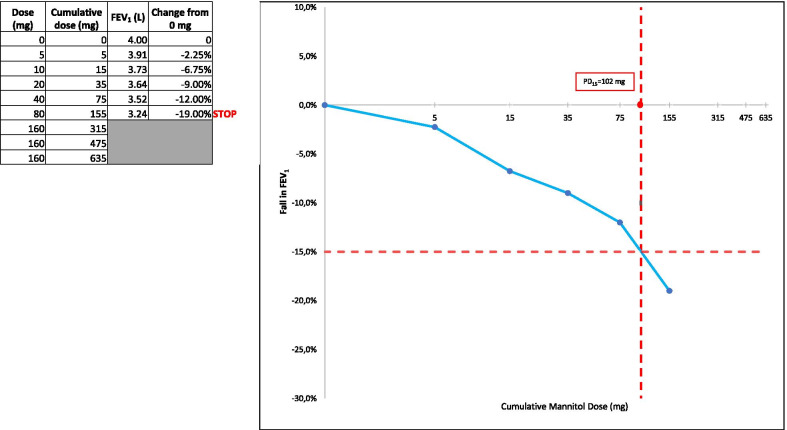
Example of mannitol test result calculations and how to report data graphically. In this case a PD_15_ of 102 mg is calculated

The PD_15_ should also be routinely reported in cases where there is a positive test. The PD_15_ provides a useful continuous measure of the extent of AHR that can also be used to assess changes over time. This is useful in monitoring patients and, of most relevance here, allows the assessment of changes in AHR in response to intervention. The following is an example of the calculation of the PD_15_: a patient that experiences a decrease in FEV_1_ of 12% at 75 mg (cumulative) and a decrease in FEV_1_ of 19% at 155 mg (cumulative), has a PD_15_ of 102 mg (Fig. 2) [13]. More information on the calculation of PD_15_ can be found in the supplementary information and this can easily be implemented using computer software.

While there are many publications focusing on the clinical use of mannitol to diagnose and manage asthma, there exists a need to assess available information on the technical and statistical aspects of using mannitol as an endpoint in clinical trials. The main objective of this review is, therefore, to assess these practical considerations and provide the clinical researcher and respiratory physician with recommendations when designing early clinical trials that utilise AHR as a marker to predict a clinical response.

This review aims to:

1. Provide an overview of the mechanism of action of the mannitol test, and a description of its advantages as an outcome measure in clinical trials
2. Examine data on repeatability of the mannitol challenge test, and variability in intervention trials, from a review of the current literature
3. Discuss what constitutes a clinically meaningful change in AHR to mannitol
4. Make recommendations on the design and analysis of asthma intervention trials using inhaled mannitol as an outcome measure, including guidance on appropriate sample sizing.

## Methods

### Literature search

A literature search of English language articles published between 1997 and 30th June 2020, in the PubMed database was conducted using the following MeSH search terms: [Asthma OR Bronchial Provocation Tests] AND [Mannitol OR Mannitol"(All Fields)]. The reference lists of all included intervention studies and identified reviews were manually searched to identify relevant studies.

### Inclusion and exclusion criteria

The authors selected papers for inclusions in this review based on the following criteria:

*Inclusion criteria* Use of standard mannitol test at multiple timepoints; therapeutic intervention or repeatability studies; reporting the use of PD_15_ as a measure.

*Exclusion criteria* Non-human trials; trials not related to asthma; reviews or commentaries; duplicate papers based on same study; no or single mannitol test included; challenge intervention (e.g., histamine) rather than therapeutic; step up/down bronchodilator trials with varying doses; non-standardised mannitol test; PD_15_ results not reported or not able to be used (e.g., not log-transformed or insufficient summary information).

### Statistical analysis and other calculations

Geometric means, 95% confidence intervals, log_2-_transformed means and standard deviations (within-subject for repeatability and crossover studies; between-subject for parallel group studies) were calculated based on PD_15_ values or change in PD_15_ values where applicable.

Undefined PD_15_ values where the FEV_1_ dropped by less than 15% at the highest cumulative dose of 635 mg were replaced with 635 mg as this is a conservative approach (see later sections for a discussion of alternative approaches). Sample size calculations were performed using standard methods for crossover and parallel group studies [16].

Raw data (where available) were used for the calculations, which were either provided from the published paper or the authors directly.

## Results

### Search results

**Fig. 3:**
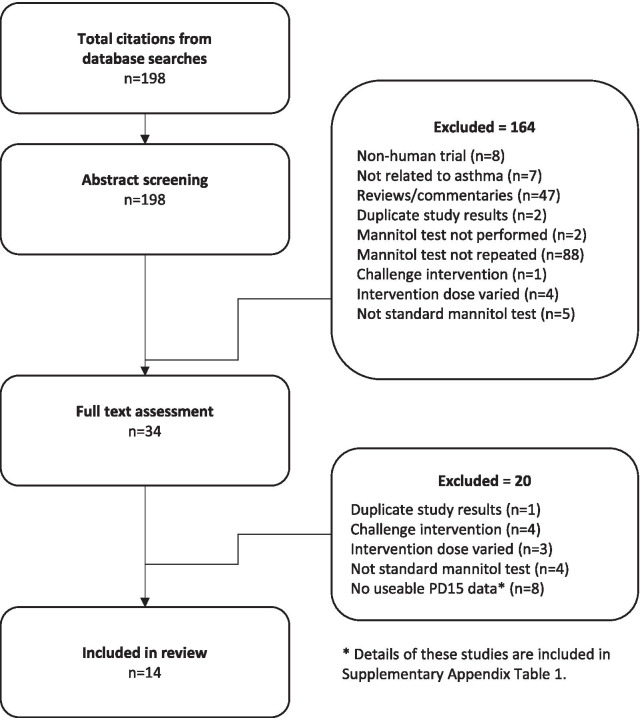
Flow chart of search strategy and study selection

A total of 193 records were retrieved from the database after implementing the search strategy. Of these, three duplicate study results were excluded as well as 178 records excluded based on other exclusion criteria. Therefore, 12 studies fulfilled the inclusion criteria (Fig. 3) and information from these studies is contained in Table 1. Additional information on these studies and also on studies that did not report PD_15_ values but met all other inclusion/exclusion criteria are contained in Additional file 1: Table S1.

**Table 1:** Summary of information from studies used in the review

Study (publication year)	n*	Screening PD_15_ inclusion criterion?	Interventions	Treatment duration (or gap between measures for repeatability)	Original scale	DD scale (log_2_ transformed)		
					Geometric mean ratio (95% CI)	Difference between treatments	Within-subject SD	Between-subject SD of change
REPEATABILITY: 2 successive PD_15_ measurements under same conditions, no interventions								ND
Barben et al. (2003) [17]	17	≤ 635	N/A	2–7 days	1.050 (0.941–1.172)	0.071	0.673	
Udesen et al. (2017)^†^ [18]	41	No	N/A	6 months	1.225 (0.907–1.652)	0.292	0.598	
CROSSOVER: 2 treatments, one post-treatment PD_15_ measurement for each treatment								
Brannan et al. (2000) [19]	24	< 350	Nedocromil/ Placebo	Single dose	2.625 (1.893–3.641)	1.392	0.791	
Brannan et al. (2001) [20]	20	< 290	Fexofenadine/ Placebo	Single dose	2.696 (1.713–4.242)	1.431	0.988	
	19	< 290	Montelukast/ Placebo	Single dose	0.825 (0.653–1.043)	− 0.278	0.496	
CROSSOVER: 2 treatments, pre and post PD_15_ measurements for each treatment								
Anderson et al. (2012)^‡^ [21]	21	No	FP 500 mg/ FP 100 mg	2 weeks	1.4 (0.7–3.1)	0.5^‡^	1.2^‡^	
Clearie et al. (2012) [22]	13	≤ 635	FP + SM/ FP (smokers)	2 weeks	1.5	0.6	1	
	11	≤ 635	FP + SM/ FP (non-smokers)		2	1	0.9	
Brannan et al. (2015) [23]	23	≤ 315	Fish oil/ placebo	3 weeks	0.76 (0.43 –1.32)	− 0.402	0.94	
PARALLEL GROUP: 2 or more treatment groups, pre- and post-treatment PD_15_ measurements								
Barakat et al. (2012) [24]	11	≤ 635	FP 100 mg	7 weeks	N/A	N/A	1.3	1.61
	11		FP 500 mg		0.62 (0.21–1.78)	− 0.7	1.0	
Toennesen et al. (2018)^†^ [25]	34	No	Control (no treatment)	8 weeks	N/A	N/A	0.87	1.36
	29		Exercise		0.81 (0.71–0.92)	0.30	1.15	
	33		Diet		0.92 (0.81–1.05)	− 0.12	0.88	
	29		Exercise + diet		0.91 (0.78–1.05)	− 0.14	0.90	
SINGLE GROUP: Comparing change in PD_15_ pre- to post-treatment								
Brannan et al. (2002) [26]	18	Yes ≤ 635	Budesonide	6–9 weeks	3.73 (2.87–4.86)	1.90	0.52	0.74
Koskela et al. (2003)^§^ [27]	17	Yes ≤ 635	Budesonide	6 months	3.6 (1.6–8.4)	1.85	1.7	2.4
Kersten et al. (2011) [28]	17	No	Dropping of LABA	30 days	0.92 (0.64–1.32)	− 0.13	0.73	1.03

Negative tests (PD_15_ > 635 mg) have a PD_15_ value of 635 mg imputed for use in calculations, except where noted

*DD*: dose doubling, *FP*: fluticasone, *LABA* long-acting beta_2_-agonists, *N/A* not applicable, *ND*: not determined, *RDR* response-dose ratio, *SD* standard deviation, *SM* salmeterol

*n = subjects used in calculation (may be a subset of total study population)

^†^PD_15_ data not published—anonymised raw data provided by authors

^‡^Approximated from plots in publication

^§^1270 imputed for negative tests rather than 635 in summary data (no raw data available)

### AHR to mannitol as an outcome measure in asthma

For AHR to be used as an outcome measure in an intervention trial, several questions need to be addressed: First, we consider the optimal way to report test results, secondly, we look at the properties of the test in terms of repeatability and other measures of variability, and what kind of changes in AHR can be feasibly detected. Finally, based on these considerations, we provide estimations of the required sample size for specific types of interventions studies, when using the response to the mannitol test as an outcome.

### Reporting mannitol test results

The simplest interpretation of a test outcome is the dichotomous *positive* (a decrease of ≥ 15% in FEV_1_ from baseline) or *negative* (a decrease of less than 15% in FEV_1_ from baseline after administration of a cumulative dose of 635 mg of inhaled mannitol). This simple dichotomisation has proved useful for diagnostic work-up of asthma.

In a large number of population studies in adults and children [29], measurements of AHR (such as PD_20_ for methacholine) have been shown to have log-normal distributions (i.e., the variability tends to increase alongside the value). Therefore, PD_15_ values are generally converted to a logarithmic scale prior to analysis which allows statistical tests that assume normality (e.g., t-tests) to be used. To aid in interpretation, it is recommended to use a logarithm to base 2 (log_2_) transformation corresponding to a dose doubling (DD) scale. A difference in log_2_(PD_15_) values of 1 on this scale equates to a doubling of the dose required for a 15% fall in FEV_1_. Using this DD scale for analysis means that a change in PD_15_ from a cumulative dose of 15 mg to 30 mg (i.e., 1 DD) is considered to be the same magnitude as a change from 315 to 630 mg as both represent a doubling in PD_15_.

All ten publications identified in the literature review that presented PD_15_ results used log-transformed values. The PD_15_ changes were examined for the eight included studies with raw data available, and while the normality assumptions were reasonably well met for the untransformed values, the log-transformed values tended to meet the assumptions slightly better. The exception was where a few very small PD_15_ readings (< 15 mg) resulted in extreme outlying values for the difference on the DD scale [20]. Lussana et al. (2015) [30] reported untransformed values (see Additional file 1: Table S1); no raw data was published. However, graphical presentations of this data suggest a skewed distribution on the untransformed scale and hence log transformation may have been beneficial. Therefore, in general, we recommend logarithmic transformation, although examination of the appropriateness of this prior to analysis is worthwhile. Analysis with untransformed data may be preferable if some patients have very small (< 15 mg) values as resulting changes on the log scale will lead to extreme, outlying values. In addition, the interpretation of results on the untransformed scale may be more intuitive and straightforward, as treatment effects can be summarised as mean differences in mg between PD_15_ values. Consideration should also be given to exclusion of patients with severe AHR (PD_15_ < 15 mg) since reliable estimation is a challenge and resulting changes can be extreme, particularly on the DD scale.

**Fig. 4:**
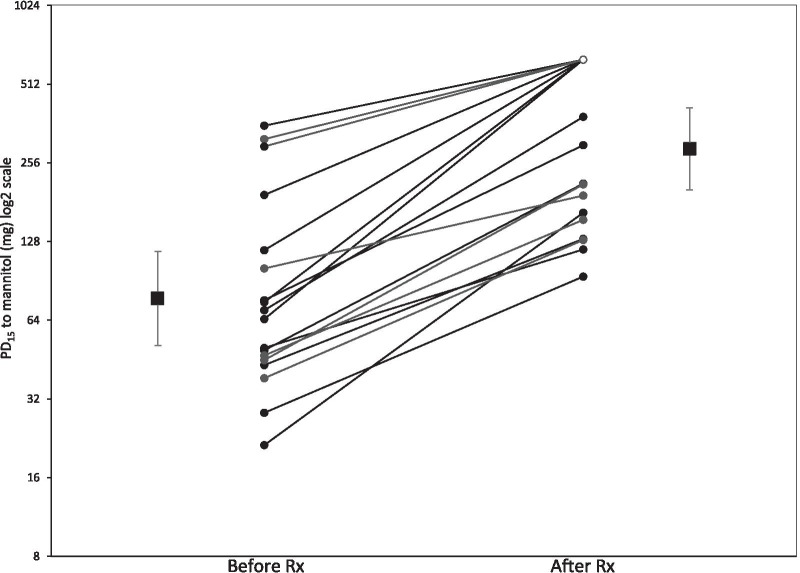
Airway responsiveness to mannitol: PD_15_ values calculated for individual patients alongside geometric means. Mean values (95% CI error bars) before and after treatment with budesonide (Rx). ○ indicates where PD_15_ values were not attained (635 mg value imputed) (adapted from Brannan et al*.* [26])

If log transformation is performed, we recommend converting back to the original scale for reporting as this is more easily interpreted. When summarising results on the original scale, geometric means (the anti-log of arithmetic means of the log-transformed values) and 95% confidence intervals are the appropriate summary measures to present. Table 2 shows estimates for the comparison between treatments both on the original scale and the DD scale. Figure 4 further shows an example of how to report data graphically.

**Table 2:** Example of results from a crossover study reporting PD_15_ on the original and dose doubling (DD) scale [26]

Subject	PD_15_ Results on original scale			Results on DD scale (log_2_ transformed data)		
	Placebo (P)	Nedocromil (N)	Ratio N:P	Placebo (P)	Nedocromil (N)	Difference (N-P)
1	188.6	379	2.01	7.559	8.566	1.007
2	36.2	≥ 635	17.54	5.178	9.311	4.133
3	76.8	569	7.41	6.263	9.152	2.889
4	> 635	> 635	1.00	9.311	9.311	0.000
5	292.4	> 635	2.17	8.192	9.311	1.119
6	210	> 635	3.02	7.714	9.311	1.596
7	234.7	> 635	2.71	7.875	9.311	1.436
8	86.6	328.6	3.79	6.436	8.360	1.924
9	561.3	> 635	1.13	9.133	9.311	0.178
10	258.8	> 635	2.45	8.016	9.311	1.295
11	128.4	285.5	2.22	7.005	8.157	1.153
12	76.8	122.8	1.60	6.263	6.940	0.677
13	104.1	122.5	1.18	6.702	6.937	0.235
14	> 635	> 635	1.00	9.311	9.311	0.000
15	106.7	> 635	5.95	6.737	9.311	2.573
16	104.4	170.3	1.63	6.706	7.412	0.706
17	38.7	499.9	12.92	5.274	8.965	3.691
18	223.2	> 635	2.84	7.802	9.311	1.508
19	124.2	368.9	2.97	6.957	8.527	1.571
20	100.2	172.8	1.72	6.647	7.433	0.786
21	24.8	122.8	4.95	4.632	6.940	2.308
22	142.2	554.5	3.90	7.152	9.115	1.963
23	630.4	> 635	1.01	9.300	9.311	0.010
24	401.9	> 635	1.58	8.651	9.311	0.660
Geometric Mean	155.8	409.1	2.63	7.284	8.676	1.392
(95% CI)	(107.0–227.1)	(317.7–526.9)	(2.56–2.69)	(6.741–7.827)	(8.311–9.041)	(1.357–1.428)

Negative tests (PD_15_ > 635 mg) have a PD_15_ value of 635 mg imputed for use in calculations

*CI* confidence interval, *DD* dose doubling, *PD*_*15*_ provoking dose at 15% drop in FEV_1_

### Dealing with undefined PD_15_ values

A potential issue with the use of PD_15_ is that it is only defined when the FEV_1_ levels drop by 15% or more, at or before the highest cumulative dose of 635 mg. Patients included in trials assessing the effectiveness of an intervention using AHR will be those who are assessed as hyperresponsive to mannitol and hence will have a PD_15_ of less than 635 mg at screening. However, FEV_1_ levels of patients with a PD_15_ at (or close to) 635 mg at screening may drop by less than 15% at the top dose on repeated testing due to random variation (repeatability issues are discussed in the next section). In addition, if an intervention is effective at reducing AHR, such patients will not have PD_15_ defined after intervention. It is therefore advisable to consider recruiting a population with moderate to severe AHR (e.g., PD_15_ < 315 mg at screening) so that even with an intervention that results in a doubling of PD_15_, the post-intervention PD_15_ is still likely to be assessable.

Data from a patient that experiences a decrease in FEV_1_ of less than 15% from baseline after administration of cumulative 635 mg of inhaled mannitol (i.e., no PD_15_ defined) can be handled in several different ways. Some authors have removed data of patients with no PD_15_ defined post-intervention from the analysis of PD_15_ altogether [17, 28, 30]. However, these patients will typically be those experiencing the largest improvements with the intervention and this may result in substantial underestimation of the treatment effect and less power. Others have replaced missing values with a PD_15_ of 1270 mg (i.e., assuming a PD_15_ of double the highest cumulative dose of the test for these patients) [21, 27]. However, if patients are experiencing falls in FEV_1_ close to (but less than) 15% at the highest dose of 635 mg this may result in a substantial over-estimation of the treatment effect.

An alternative approach, (used by Brannan et al. [19] and Brannan et al*.* (2002) [26]) that neither excludes patients from analysis nor potentially overestimates treatment effect, is to impute a value of 635 mg for those not achieving a PD_15_ during the test. This will be, by definition, an underestimation of the true PD_15_ that would be achieved if dosing were to continue. However, if used in conjunction with limiting the patient population to subjects with a starting PD_15_ of < 315 mg, using 635 mg represents at least a doubling of PD_15_ post-intervention, and can be considered a lower bound of the effect in these patients. Finally, a different approach would be to linearly extrapolate on the log dose scale using the last and the second-last cumulative dose of inhaled mannitol, and the corresponding falls in FEV_1_ values in case of a negative test. As the relationship between percent fall in FEV_1_ and log dose does not appear to be linear, this will potentially tend to over-estimate the PD_15_. This approach has not been used in the reviewed literature.

We have restricted our focus to PD_15_ in this paper, as this is the measure of the degree of AHR used in clinical practice. The response-dose ratio (RDR) has been proposed as an alternative outcome measure from the mannitol test that is defined even when the PD_15_ is not reached and is commonly used in epidemiology settings where healthy subjects are included. The RDR is calculated by taking the final percent fall in FEV_1_ (from the highest cumulative dose administered during the mannitol test) and dividing it by the cumulative dose that induced that percent fall. PD_15_ is recommended where patients with scope for improvement in AHR are recruited, particularly as the RDR for a patient does not appear to be constant across the dose range of the mannitol test.

To illustrate the discussed reporting concepts, Table 2 shows the PD_15_ results from Brannan et al. [26] which examined the effect of nedocromil on AHR using the mannitol test. This was a 2-period crossover study of a single dose of nedocromil versus placebo. Those subjects who did not record a 15% decrease in FEV_1_ at the highest dose of 635 mg were assigned a PD_15_ of 635 mg for inclusion in the calculations in the paper (which is in line with our recommended approach). Consequently, the results should be interpreted as a conservative estimate of the true effect of nedocromil on AHR. This study found a difference of 1.4 on the DD scale which equates to a 2.6-fold increase on the original untransformed scale.

### Repeatability of the mannitol test

In order for an outcome measure to be considered a suitable endpoint for use in a clinical trial design it is important to first assess the repeatability of the test. Repeatability is defined as the closeness of the agreement between the results of successive measurements (in this case PD_15_ measurements) carried out under similar conditions. A test with poor repeatability will not be appropriate for the measurement of intervention-related changes since these changes would be difficult to distinguish from the natural variability of the test results.

Two studies [17, 18] in the literature review were identified as having relevant information on the repeatability of PD_15_ measurements under the same conditions with varying time periods (up to 6 months) between the measurements (Table 1). The difference on the DD scale is close to 0 in both studies indicating little systematic change over time in the PD_15_ measurement in the absence of an intervention. The within-subject variability on the DD scale was similar in both studies at around 0.6. The intraclass correlation was 91% and 83% in the two repeatability studies, indicating good repeatability of measures within a subject when compared to the overall variability of test results.

### What is a meaningful change in AHR to mannitol?

An important consideration before applying a new outcome measure is its ability to detect changes in AHR after an intervention that correspond to clinically meaningful efficacy. Responsiveness to indirect agents is significantly reduced after inhaled corticosteroids (ICS) treatment within weeks to months, and changes in AHR measured by the mannitol test after ICS treatment correlate with the changes in symptom scores and FEV_1_ and is also related to less frequent use of short-acting beta_2_-agonists [26, 27]. Furthermore, an increase in AHR to inhaled mannitol has proven to be as sensitive as sputum eosinophils as an indicator of loss of disease control through down-titration of ICS, and better than perceived symptoms, fraction of exhaled nitric oxide or lung function [31]. Finally, a study conducted in primary care using AHR to mannitol to adjust ICS treatment resulted in fewer mild exacerbations as well as a reduction in exhaled nitric oxide, symptoms and reliever use compared with a control group using lung function and symptom scores to adjust treatment [32]. These findings indicate that changes in AHR to inhaled mannitol are related to changes in parameters considered important in evaluating clinical efficacy of an intervention.

The results from the intervention studies using AHR as an outcome measure (Table 1) can also help in the planning of future studies (see later section on sample size calculations), based on the expected effect size of a new treatment compared to existing and already tested interventions like ICS, antihistamines or montelukast.

When compared to placebo, the reported improvements in PD_15_ to inhaled mannitol as a result of effective interventions (such as nedocromil, fexofenadine and budesonide) are above a twofold increase in PD_15_ (equivalent to a difference of 1 on the DD scale; Table 1). Based on this, as well as the fact that the within-subject variability in PD_15_ on the log_2_ scale is around 0.6 across the studies, we suggest using 1.0 on the DD scale (i.e., a doubling on the original scale) as a cut-off for a meaningful change in AHR to mannitol.

### Sample size calculations for trials using AHR to inhaled mannitol as the outcome

A sample size calculation is one of the core elements of a well-designed trial. The appropriate number of patients needed in a clinical study can be calculated from the anticipated standard deviation, the minimum clinically important difference (MCID) and the desired type 1 error (α) and power (1-β) [16].

Numerous online calculators now provide easy access to sample size calculations. Table 3 provides worked examples on the number of patients needed to determine whether an intervention has an impact on AHR depending on the study design. For simplicity, we have also assumed that an intervention will be compared against placebo, although the same considerations would apply for comparison against an active control. We have also assumed β = 0.2 (corresponding to 80% power) and 2-sided α of 0.05. Based on the data presented in the previous section the MCID was set to 1.0 DD.

**Table 3:** Worked examples for sample size calculations—based on choice of study design, existing literature on MCID and within- and between-subject SD using PD_15_ to inhaled mannitol as outcome measure

	Crossover study design—single PD_15_ measure	Crossover study design –pre/post PD_15_ measures	Parallel group design—pre/post PD_15_ measures
Recommended if	Single dose of drug	Short duration of intervention	Longer duration of intervention
Outcome measure	PD_15_ post-treatment	Change in PD_15_ pre/post-treatment	Change in PD_15_ pre/post-treatment
Within-subject SD*	0.8	–	–
SD* of within-subject difference	–	1.13	–
Between-subject SD* of differences	–	–	1.4
MCID	1 on DD scale (i.e., doubling in PD_15_)	1 on DD scale (i.e., doubling in PD_15_)	1 on DD scale (i.e., doubling in PD_15_)
Total Sample size needed^†^	13	23	64

*DD* dose doubling, *MCID* minimum clinically important difference, *PD*_*15*_ provoking dose at 15% drop in FEV_1_; *SD* standard deviation

*All SDs are on DD scale i.e., log_2_ transformed data; ^†^With a probability of a type-I error of 2-sided 5% and power of 80%

The appropriate standard deviation (SD) to use (within-subject SD or between-subjects SD of PD_15_ measurements) depends on the design of the study. If the intervention is either a single dose of a drug such as nedocromil (e.g., in Brannan et al. [19]) or a short duration of treatment (up to a few weeks, e.g., fish oil in Brannan et al. [23]), then a crossover design where each patient receives both the intervention and placebo is optimal and an estimate of the within-subject SD of PD_15_ is the appropriate SD (Table 1). Where a pre- and post-treatment PD_15_ result is recorded for each treatment, the SD of the differences which is $\surd 2$ times the within-subject SD would be appropriate. The within-subject SD appears higher in intervention studies compared to the repeatability studies (Table 1). This is not surprising as additional variability is introduced due to person-to-person variations in the effects of the intervention. Alternatively, if the study design is a parallel group design where patients are randomised to two groups, and each group receives either the intervention of interest or placebo (or comparator intervention) then the appropriate SD to use in the calculation will be the between-subject SD of changes in PD_15_ measurements.

In general, when determining the appropriate sample size for a new study, we recommend using SD estimates from a similar prior study, in terms of design and duration of treatment, as well as estimated treatment effect (e.g., fexofenadine versus budesonide).

## Conclusions

Measuring AHR to inhaled mannitol is currently being used as a diagnostic tool in asthma. The high specificity of the test illustrates the close association with ongoing airway inflammation in terms of mast cell infiltration with or without eosinophilia. The mannitol test holds several practical advantages in its standardisation and efficiency, with just one test protocol and no requirement for a nebuliser or equipment other than a spirometer, making it ideal in a clinical trial setting. These mechanistic and practical features of the mannitol test make it a useful potential marker of disease that is not only suitable for diagnosing disease clinically, but as an outcome measure in intervention trials.

Our findings should be considered in light of the following limitations. Firstly, the review only identified English language publications in peer-reviewed journals held in the PubMed database. Secondly, only those studies using PD_15_ as a measure were included, although this is considered a measure of the degree of AHR most commonly used in clinical practice. There were few studies specifically looking at the repeatability of PD_15_, particularly in settings relevant to the use of this measure in trials of new agents. In addition, there were relatively few studies, with a small number of subjects in each, where the standard deviations needed for sample sizing in future studies could be estimated.

We have summarised existing data on serial measurements of AHR to inhaled mannitol in either repeatability or intervention trials to describe the usefulness of the mannitol test as an outcome measure in clinical trials. Based on our findings, we suggest AHR to inhaled mannitol is reported as the provoking dose that causes a 15% fall in FEV_1_ (PD_15_) and that changes in AHR are generally reported in doubling doses. We further suggest that subjects that turn negative to the test after an intervention will be handled as having a PD_15_ of 635 mg, keeping in mind that in doing so the impact of the intervention may be underestimated. However, if inclusion of patients into a trial is limited to those with a PD_15_ < 315 mg at baseline, a PD_15_ of 635 mg post-intervention represents at least a doubling of the PD_15_ prior to intervention. Bearing in mind we also show that the within-subject variability in PD_15_ on the log_2_ scale is around 0.6 across studies, a change of 1.0 DD can be considered unlikely to be a result of test-to-test variation.

There have been no studies performed with the sole purpose of defining what a minimal clinically important difference in PD_15_ is. However, based on the existing studies reporting parallel changes in symptom scores, lung function, use of PRN medications and changes in AHR due to various interventions, we suggest using a MCID of 1.0 DD as this has proven to be associated with significant changes in patient-related outcomes.

Finally, we provide examples on sample size calculations for the planning of future trials. It is striking to note, that due to the reproducibility of the mannitol test and the effect size of regular anti-inflammatory treatments on AHR to mannitol, the number of patients needed to show a meaningful effect on AHR to mannitol is relatively low. Altogether, these data suggest that measuring AHR to mannitol provides a novel and reproducible test for assessing efficacy in intervention trials, and importantly, utilises a test that links directly to underlying drivers of disease. In future, AHR to mannitol may provide a tool for identifying responders to new treatments that include targeting mast cell driven airway disease.

## Supplementary Information

**Additional file 1: Table S1.** Summary of information from the 12 studies included in the review and 7 studies excluded because of no usable PD_15_ data.

## Data Availability

Data sharing is not applicable to this article as no datasets were generated during the current study. The raw data that support the findings from the studies described in the Toennesen et al*.* [25] and Udesen et al*.* [17] papers were provided by the authors and requests for access should be directed to them.

## Abbreviations

AHR: Airway hyperresponsiveness
AMP: Adenosine monophosphate
BPT: Bronchial provocation tests
DD: Dose doubling
EMA: European Medicines Agency
EVH: Eucapnic voluntary hyperventilation test
FEV_1_: Forced expiratory volume in the first second
FDA: Food and Drug Administration
FP: Fluticasone
ICS: Inhaled corticosteroids
LABA: Long-acting beta2-agonist
MCID: Minimum clinically important difference
MeSH: National Library of Medicine’s (NLM’s) controlled vocabulary or subject heading list
N/A: Not applicable
ND: Not determined
PD: Provoking dose
PD_15_: Dose that provokes a 15% drop in FEV_1_
PRN: Taken on an as required basis
PICOS: Participants, interventions, comparisons, outcomes, and study
RDR: Response–dose ratio
SD: Standard deviation
SM: Salmeterol

## References

[1] Agustí A, Bafadhel M, Beasley R, Bel EH, Faner R, Gibson PG (2017). Precision medicine in airway diseases: moving to clinical practice. Eur Respir J.

[2] Pavord ID, Holliday M, Reddel HK, Braithwaite I, Ebmeier S, Hancox RJ (2020). Predictive value of blood eosinophils and exhaled nitric oxide in adults with mild asthma: a prespecified subgroup analysis of an open-label, parallel-group, randomised controlled trial. Lancet Respir Med.

[3] Backer V, Sverrild A, Ulrik CS, Bødtger U, Seersholm N, Porsbjerg C (2015). Diagnostic work-up in patients with possible asthma referred to a university hospital. Eur Clin Respir J..

[4] European Medicines Agency. Guideline on the clinical investigation of medicinal products for the treatment of asthma (CHMP/EWP/2922/01 Rev. 1). https://www.ema.europa.eu/en/documents/scientific-guideline/guideline-clinical-investigation-medicinal-products-treatment-asthma_en.pdf (2015). Accessed 25 March 2021.

[5] Prosperini G, Rajakulasingam K, Cacciola RR, Spicuzza L, Rorke S, Holgate ST (2002). Changes in sputum counts and airway hyperresponsiveness after budesonide: monitoring anti-inflammatory response on the basis of surrogate markers of airway inflammation. J Allergy Clin Immunol.

[6] van Den Berge M, Meijer RJ, Kerstjens HA, de Reus DM, Koëter GH, Kauffman HF (2001). PC(20) adenosine 5'-monophosphate is more closely associated with airway inflammation in asthma than PC(20) methacholine. Am J Respir Crit Care Med.

[7] Sverrild A, Porsbjerg C, Backer V (2012). The use of inhaled mannitol in the diagnosis and management of asthma. Expert Opin Pharmacother.

[8] Kernen P, Steveling-Klein EH, Saccilotto RT, Raatz H, Briel M, Koller MT (2019). The sensitivity and specificity of the mannitol bronchial challenge test to identify asthma in different populations: a systematic review. Swiss Med Wkly..

[9] Porsbjerg C, Brannan JD, Anderson SD, Backer V (2008). Relationship between airway responsiveness to mannitol and to methacholine and markers of airway inflammation, peak flow variability and quality of life in asthma patients. Clin Exp Allergy.

[10] Sverrild A, Bergqvist A, Baines KJ, Porsbjerg C, Andersson CK, Thomsen SF (2016). Airway responsiveness to mannitol in asthma is associated with chymase-positive mast cells and eosinophilic airway inflammation. Clin Exp Allergy.

[11] Porsbjerg C, Sverrild A, Backer V (2015). Combining the Mannitol Test and FeNO in the Assessment of Poorly Controlled Asthma. J Allergy Clin Immunol Pract..

[12] Anderson SD, Brannan J, Spring J, Spalding N, Rodwell LT, Chan K (1997). A new method for bronchial-provocation testing in asthmatic subjects using a dry powder of mannitol. Am J Respir Crit Care Med.

[13] Anderson SD (2010). Indirect challenge tests: Airway hyperresponsiveness in asthma: its measurement and clinical significance. Chest.

[14] Brannan JD, Lougheed MD (2012). Airway hyperresponsiveness in asthma: mechanisms, clinical significance, and treatment. Front Physiol.

[15] Brannan JD, Anderson SD, Perry CP, Freed-Martens R, Lassig AR, Charlton B (2005). The safety and efficacy of inhaled dry powder mannitol as a bronchial provocation test for airway hyperresponsiveness: a phase 3 comparison study with hypertonic (4.5%) saline. Respir Res..

[16] Julious SA (2004). Sample sizes for clinical trials with Normal data2004 in Statistics in Medicine. Statist Med.

[17] Barben J, Roberts M, Chew N, Carlin JB, Robertson CF (2003). Repeatability of bronchial responsiveness to mannitol dry powder in children with asthma. Pediatr Pulmonol.

[18] Udesen PB, Westergaard CG, Porsbjerg C, Backer V (2017). Stability of FeNO and airway hyperresponsiveness to mannitol in untreated asthmatics. J Asthma.

[19] Brannan JD, Anderson SD, Freed R, Leuppi JD, Koskela H, Chan HK (2000). Nedocromil sodium inhibits responsiveness to inhaled mannitol in asthmatic subjects. Am J Respir Crit Care Med.

[20] Brannan JD, Anderson SD, Gomes K, King GG, Chan HK, Seale JP (2001). Fexofenadine decreases sensitivity to and montelukast improves recovery from inhaled mannitol. Am J Respir Crit Care Med.

[21] Anderson WJ, Short PM, Williamson PA, Lipworth BJ (2012). Inhaled corticosteroid dose response using domiciliary exhaled nitric oxide in persistent asthma: the FENOtype trial. Chest.

[22] Clearie KL, McKinlay L, Williamson PA, Lipworth BJ (2012). Fluticasone/Salmeterol combination confers benefits in people with asthma who smoke. Chest.

[23] Brannan JD, Bood J, Alkhabaz A, Balgoma D, Otis J, Delin I (2015). The effect of omega-3 fatty acids on bronchial hyperresponsiveness, sputum eosinophilia, and mast cell mediators in asthma. Chest.

[24] Baraket M, Oliver BG, Burgess JK, Lim S, King GG, Black JL (2012). Is low dose inhaled corticosteroid therapy as effective for inflammation and remodeling in asthma? A randomized, parallel group study. Respir Res.

[25] Toennesen LL, Meteran H, Hostrup M, Wium Geiker NR, Jensen CB, Porsbjerg C (2018). Effects of exercise and diet in nonobese asthma patients-a randomized controlled trial. J Allergy Clin Immunol Pract.

[26] Brannan JD, Koskela H, Anderson SD, Chan HK (2002). Budesonide reduces sensitivity and reactivity to inhaled mannitol in asthmatic subjects. Respirology.

[27] Koskela HO, Hyvärinen L, Brannan JD, Chan HK, Anderson SD (2003). Sensitivity and validity of three bronchial provocation tests to demonstrate the effect of inhaled corticosteroids in asthma. Chest.

[28] Kersten ET, Driessen JM, Duiverman EJ, Thio BJ (2011). The effect of stepping down combination therapy on airway hyperresponsiveness to mannitol. Respir Med.

[29] Peat JK, Gray EJ, Mellis CM, Leeder SR, Woolcock AJ (1994). Differences in airway responsiveness between children and adults living in the same environment: an epidemiological study in two regions of New South Wales. Eur Respir J.

[30] Lussana F, Di Marco F, Terraneo S, Parati M, Razzari C, Scavone M (2015). Effect of prasugrel in patients with asthma: results of PRINA, a randomized, double-blind, placebo-controlled, cross-over study. J Thromb Haemost.

[31] Leuppi JD, Salome CM, Jenkins CR, Anderson SD, Xuan W, Marks GB (2001). Predictive markers of asthma exacerbation during stepwise dose reduction of inhaled corticosteroids. Am J Respir Crit Care Med.

[32] Lipworth BJ, Short PM, Williamson PA, Clearie KL, Fardon TC, Jackson CM (2012). A randomized primary care trial of steroid titration against mannitol in persistent asthma: STAMINA trial. Chest.

