# Supplementary material for: The use of the mannitol test as an outcome measure in asthma intervention studies: a review and practical recommendations
Source: Respir Res. 2021 Nov 7;22:287. doi: 10.1186/s12931-021-01876-9 (PMC8574016; doi:10.1186/s12931-021-01876-9)
Supplement: Supplementary file 1 — Additional file 1: Table S1. Summary of information from the 12 studies included in the review and 7 studies excluded because of no usable PD15 data. [file 12931_2021_1876_MOESM1_ESM.pdf]

Additional File 1

The use of the mannitol test as an outcome measure in asthma intervention studies: a review and practical recommendations

**Journal:**	Respiratory Research
**Manuscript ID:**	Supplementary Appendix
**Article Type:**	Original article - Review
**Complete List of Authors:**	Sverrild A, Leadbetter J, Porsbjerg C

Supplementary Information:

PD_{15} calculation……………………………………………………………..Page 2

Table S1. Summary of information from the 14 studies included in the review and 8 studies excluded because of no usable PD_{15} data …………………………Page 3

Table S2. Example of results from a crossover study reporting PD_{15} on the original and dose doubling (DD) scale ……………………………………………………..Page 8

Practical Considerations for the conduct of the Mannitol Challenge Test……..Page 9

PD_{15} calculation

The calculation for PD_{15} based on linear interpolation on the $\log_2$ scale is given below:

$$\log_2(PD_{15}) = \log_2(d_{j-1}) + \frac{(\log_2(d_j) - \log_2(d_{j-1})) * (15 - (\%\, FEV_1\, fall\ at\ d_{j-1}))}{(\%\, FEV_1\, fall\ at\ d_j) - (\%\, FEV_1\, fall\ at\ d_{j-1})}$$

$Where\ d_j\ is\ the\ last\ cumulative\ dose, and\ d_{j-1}\ is\ the\ second\ last\ cumulative\ dose.$

Notes:

- We have used a $\log_2$ transformation here, consistent with the use of $\log_2$ transformation of the PD_{15} results in the analysis. However, the calculation is unaffected by the use of other bases, e.g., $\log_{10}$ or $\log_e$
- In rare cases FEV_1 may fall by ≥ 15% at the first dose of 5 mg, and hence the second-last cumulative dose is 0mg which cannot be log-transformed. A small value, such as 0.001mg, should be substituted as the second-last cumulative dose in the above formula to allow the calculation to proceed. (Since the PD_{15} estimation is volatile for these subjects and any small increase due to intervention will result in an outlying high fold change, consideration should be given to not continuing such a subject in the trial)
- In rare cases, the mannitol test may be stopped because of a > 10% fall in FEV1 between successive non-zero doses but FEV_1 has not dropped by ≥ 15% from baseline then the PD_{15} cannot be calculated by interpolation. In order to not have missing PD_{15} data for these cases, extrapolation may be considered using the following formula:

$$\log_2(PD_{15}) = \log_2(d_j) + \frac{(\log_2(d_j) - \log_2(d_{j-1})) * (15 - (\%\, FEV_1\, fall\ at\ d_j))}{(\%\, FEV_1\, fall\ at\ d_j) - (\%\, FEV_1\, fall\ at\ d_{j-1})}$$

$Where\ d_j\ is\ the\ last\ cumulative\ dose, and\ d_{j-1}\ is\ the\ second\ last\ cumulative\ dose$

If this formula results in a PD_{15} value of > 635 mg, a value of 635 mg should be imputed.

Table S1. Summary of information from the 14 studies included in the review and 8 studies excluded because of no usable PD_{15} data.

Study (publication year)	Design	Population - key characteristics	Screening PD_{15} inclusion criterion	n*	ICS use allowed?	Interventions	Duration (of treatment or gap for repeatability)	Handling of missing PD_{15}	Source of results	Original scale: geometric mean ratio (95% CI)	DD scale: difference	DD scale: within-subject SD	Between-subject SD of change[†]
**Studies included in review**													
Barben *et al.* (2003)[1]	Repeatability	9-16 year old stable moderate/severe asthmatics, screening $ppFEV_1$ > 65%	≤ 635	17	Yes (all subjects taking)	N/A	2-7 days	Removed from analysis (replaced with 635 in recalculations)	Calculations from raw data in paper	1.050 (0.687, 1.606)	0.071	0.673	
Udesen *et al.* (2017)[2]	Repeatability	18-60 year old non-smokers, with current symptoms and either reversibility > 12%, day-to-day FEV_1 variation > 20%, positive methacholine challenge	No	41	No	N/A	6 months	N/A - focused on RDR (replaced with 635 in recalculations)	Calculations from raw data in paper	1.225 (0.907, 1.652)	0.292	0.598	
Brannan *et al.* (2000)[3]	Crossover - single dose	15-46 year old non-smokers, screening $ppFEV_1$ ≥ 75%	< 350	24	Yes (13/24 subjects taking)	Nedocromil/ placebo	Single dose	Removed from PD_{15} analysis (replaced with 635 in recalculations)	Calculations from raw data in paper	2.625 (1.893, 3.641)	1.392	0.791	
Brannan *et al.* (2001)[4]	Crossover - single dose	18-41 year old atopic non-smokers, screening $ppFEV_1$ > 70%	< 290	20	Yes	Fexofenadine/ placebo	Single dose	N/A - none missing	Calculations from raw data in paper	2.696 (1.713, 4.242)	1.431	0.988	
				19		Montelukast/ placebo	Single dose	N/A - none missing		0.825 (0.653,1.043)	-0.278	0.496	
Anderson *et al.* (2012)[5]	Crossover with pre/ post measurements	mild-moderate persistent adult asthmatics, FeNO > 30 ppb, increased by 10 following ICS washout, previous ICS 200 to 1000 BDP equivalent, non-smoker	No	21	Yes (trial treatment) - all subjects washed out of prior ICS	FP 100, FP 500 (no control)	2 weeks	Replaced with 1270	Approximated using back calculations from confidence intervals on graphs	1.4 (0.7 - 3.1)	0.5	1.2	1.7

Study (publication year)	Design	Population - key characteristics	Screening PD_{15} inclusion criterion	n*	ICS use allowed?	Interventions	Duration (of treatment or gap for repeatability)	Handling of missing PD_{15}	Source of results	Original scale: geometric mean ratio (95% CI)	DD scale: difference	DD scale: within-subject SD	Between-subject SD of change†
Clearie *et al.* (2012)[6]	Crossover with pre/post measurements	Smokers: mild-moderate persistent adult asthmatics, $ppFEV_1 \geq 60$, < 30% PEF variability, ICS dose ≤ 1000 BDP equivalent, PC 20 meth < 4	≤ 635	13	Yes (trial treatment) - all subjects washed out of prior ICS	FP/SM, FP	2 weeks	Ignored in primary analysis	Summary information in paper used	1.5	0.6	1	1.4
		Non-smokers: mild-moderate persistent adult asthmatics, $ppFEV_1 \geq 60$, < 30% PEF variability, ICS dose ≤ 1000 BDP equivalent, PC 20 meth < 4		11						2	1	0.9	1.3
Brannan *et al.* (2015)[7]	Crossover with pre/post measurements	19-54 year old non-smokers with current symptoms, screening $PD_{15} \leq 315$, $ppFEV_1$ > 70%	≤ 315	23	Yes	Fish oil	3 weeks	N/A - none missing	Calculations from raw data in paper	0.76 (0.43 - 1.32)	-0.402	0.94	1.33
Barakat *et al.* (2012)[8]	Parallel group	19-30 year old, intermittent/mild persistent/moderate persistent atopic asthmatics, symptoms in last 12 months, $ppFEV_1$ > 60%, non-smoker, no ICS or systemic corticosteroid in last 2 months	≤ 635	11	Yes (trial treatment)	FP 100	7 weeks	N/A - Focused on RDR	Summary information in paper used	N/A	N/A	1.3	1.61
				11		FP 500				0.62 (0.21-1.78)	-0.7	1.0	
Toennesen *at al.* (2018)[9]	Parallel group	18-65 year olds, BMI 20-30kg/m^2, ACQ ≥ 1, 1 positive diagnostic test, on stable treatment (ICS, ICS+beta2 agonist, LTRA) OR no prophylactic treatment	No	31	Yes (~ 60% of subjects taking)	Control	8 weeks	N/A - focused on RDR (replaced with 635 in recalculations)	Calculations from raw data provided by authors	N/A	N/A	0.87	1.36
				28		Exercise				0.81 (0.71 -0.92)	-0.30	1.15	
				28		Diet				0.92 (0.81 -1.05)	-0.12	0.88	
				21		Exercise + Diet				0.91 (0.78 -1.05)	-0.14	0.90	

Study (publication year)	Design	Population - key characteristics	Screening PD_{15} inclusion criterion	n*	ICS use allowed?	Interventions	Duration (of treatment or gap for repeatability)	Handling of missing PD_{15}	Source of results	Original scale: geometric mean ratio (95% CI)	DD scale: difference	DD scale: within-subject SD	Between-subject SD of change†
Diver al.(2021)[)#10]	Parallel group	18-75 year old, receiving medium or high dose ICS for >12 months plus ≥1 additional controller medication, $ppFEV_1$>50%, >12% FEV_1reversibility	No	24	Yes (required)	Placebo	20 weeks	Unclear	Summary information in paper used	N/A	N/A	0.94#	1.38#
				24		Tezepelumab				1.79 (1.03-3.14)	0.84		
Sverrild *et al*.(2021)[†11]	Parallel group	18-75 year olds, non-smokers, uncontrolled (ACQ-6>1), $ppFEV_1$≥	≤315	19	Yes (required)	Placebo	12 weeks	Replaced with 635	Calculations from raw data provided by authors	N/A	N/A	1.39	1.54
				20		Tezepelumab				1.9 (0.9 - 3.7)	0.9	0.67	
Brannan *et al.* (2002)[12]	Before/after	19-50 year old non-smokers, screening $ppFEV_1$ >60%	≤ 635	18	N/A (study treatment)	Budesonide 6-9 weeks	6-9 weeks	Replaced with 635	Calculations from raw data in paper	3.73 (2.87, 4.86)	1.90	0.52	0.74
Koskela *et al.* (2003)[13‡]	Before/after	43-58 year old newly diagnosed steroid-naive asthmatics with no emphysema, smokers and non-smokers, $ppFEV_1$ ≥ 50%	≤ 635	17	N/A (study treatment)	Budesonide	6 months	replace with 1270, focused on RDR	Summary information in paper used	3.6 (1.3 - 8.4)	1.85	1.7	2.4
Kersten *et al.* (2011)[14]	Before/after	12-17 year olds, mild-moderate clinically stable asthma, receiving LABA+ICS combination therapy	No	17	Yes, by design all taking LABA/ICS combination	Dropping of LABA	30 days	Ignored in primary (replaced with 635 in recalculations)	Calculations from raw data in paper	0.92 (0.64, 1.32)	-0.13	0.73	1.03

Study (publication year)	Design	Population - key characteristics	Screening PD_{15} inclusion criterion	n*	ICS use allowed?	Interventions	Duration (of treatment or gap for repeatability)	Handling of missing PD_{15}	Source of results	Original scale: geometric mean ratio (95% CI)	DD scale: difference	DD scale: within-subject SD	Between-subject SD of change†
**Studies excluded from review**													
Currie *et al.* (2003)[15]	Repeatability	Mild-to-moderate persistent atopic asthmatics, $ppFEV_1$ > 60	No	15	Yes (11/15)	N/A	3-14 days	Removed from analysis	No data could be used - plots only				
McClean *et al.* (2011)[16]	Repeatability	Non-smoking asthmatics and healthy non-asthmatics between 17-70 years of age. Asthmatics with $ppFEV_1$ > 60% or 1.4 L.	No	67	Yes (43/52 asthmatics taking)	N/A	2 weeks	N/A Focused on RDR	No data could be used - reported outcome RDR				
Currie *et al.* (2003)[17]	Crossover - single dose	21 - 66 year old mild-to-moderate persistent atopic asthmatics, PD_{20} ≤ 315, AMP PC_{20} ≤ 200 mg/ml, screening ppFEV1>60%	(PD_{20} ≤ 315)	15	Yes (12/15 subjects taking)	Montelukast/ montelukast + desloratadine /Placebo	single dose	N/A - none missing	No data could be used - PD_{20} reported not PD_{15}				
Koskela *et al.* (2005)[18]	Crossover - single dose	15-46 year old, non-smokers, $ppFEV_1$ ≥ 75%	≤315	24	Yes	Nedocromil	single dose	N/A Focused on RDR	No data could be used -RDR reported not PD_{15}				
Jabbal *et al.* (2017)[19]	Crossover with pre/post measurements	Adult non-smokers with persistent asthma already receiving ICS or ICS/LABA, $ppFEV_1$ > 50%	< 635	14	Yes, by design all taking half usual ICS dose	Indacaterol + tiotropium vs indacaterol alone	4 weeks	N/A none missing	No data could be used - no change data reported				
Lussana *et al.* (2015)[20]	Crossover with pre/post measurements	Mild/stable asthmatics aged 18-74 years without chronic medication except inhaled beta2-agonists prn or low dose ICS	≤ 635	24	No information	Prasugrel	15 days	N/A none missing	No data could be used - no individual data and PD_{15} analysed on untransformed scale				

Study (publication year)	Design	Population - key characteristics	Screening PD_{15} inclusion criterion	n*	ICS use allowed?	Interventions	Duration (of treatment or gap for repeatability)	Handling of missing PD_{15}	Source of results	Original scale: geometric mean ratio (95% CI)	DD scale: difference	DD scale: within-subject SD	Between-subject SD of change†
Lipworth *et al* (2021)[21]	Crossover with pre/post measurements	Adult smokers with persistent asthma	≤ 635	9	Yes, all current ICS users	Olodaterol/Tiotropiom/Clenil vs Olodaterol/ Clenil	2-4 weeks	N/A Different outcome	No data could be used – reported outcome $PD_{30}R5$				
Torok *et al.* (2014)[22]	Before/after	9 -20 year old asthmatics	No (inclusion based on exercise challenge test)	14	Yes (trial treatment) - all subjects no ICS within 1 month of entry	Budesonide, Montelukast	14 days (bud), last 7 days montelukast	Focused on RDR for those not failing	No data could be used - reported outcome RDR				

*n=subjects included in main analysis in the publication. †Calculated as √2 x within-subject SD. ‡ SDs using 1270 not 635 #Approximated from model results in publication
ACQ: Asthma Control Questionnaire; BDP: beclometasone dipropionate; FEV_1: forced expiratory volume in the first second; FeNO: exhaled nitric oxide; FP: fluticasone; ICS: inhaled corticosteroids; LABA: long-acting beta2-agonist; LTRA: leukotriene receptor antagonists; Meth: methacholine; N/A: not applicable; PC: provoking concentration; PD: provoking dose; PD_{15}: dose that provokes a 15% drop in FEV_1; $PD_{30}R5$: dose that provokes a 30% increase in resistance at 5Hz; PEF: peak expiratory flow; pp: percent predicted; ppb: parts per billion; RDR: response-dose ratio; SD: standard deviation; SM: salmeterol.

Table S2. Example of results from a crossover study reporting PD_{15} on the original and dose doubling (DD) scale [12].

	PD_{15} Results on original scale			Results on DD scale (log_2-transformed data)		
**Subject**	**Placebo (P)**	**Nedocromil (N)**	**Ratio N:P**	**Placebo (P)**	**Nedocromil (N)**	**Difference (N-P)**
**1**	188.6	379	2.01	7.559	8.566	1.007
**2**	36.2	≥ 635	17.54	5.178	9.311	4.133
**3**	76.8	569	7.41	6.263	9.152	2.889
**4**	> 635	> 635	1.00	9.311	9.311	0.000
**5**	292.4	> 635	2.17	8.192	9.311	1.119
**6**	210	> 635	3.02	7.714	9.311	1.596
**7**	234.7	> 635	2.71	7.875	9.311	1.436
**8**	86.6	328.6	3.79	6.436	8.360	1.924
**9**	561.3	> 635	1.13	9.133	9.311	0.178
**10**	258.8	> 635	2.45	8.016	9.311	1.295
**11**	128.4	285.5	2.22	7.005	8.157	1.153
**12**	76.8	122.8	1.60	6.263	6.940	0.677
**13**	104.1	122.5	1.18	6.702	6.937	0.235
**14**	> 635	> 635	1.00	9.311	9.311	0.000
**15**	106.7	> 635	5.95	6.737	9.311	2.573
**16**	104.4	170.3	1.63	6.706	7.412	0.706
**17**	38.7	499.9	12.92	5.274	8.965	3.691
**18**	223.2	> 635	2.84	7.802	9.311	1.508
**19**	124.2	368.9	2.97	6.957	8.527	1.571
**20**	100.2	172.8	1.72	6.647	7.433	0.786
**21**	24.8	122.8	4.95	4.632	6.940	2.308
**22**	142.2	554.5	3.90	7.152	9.115	1.963
**23**	630.4	> 635	1.01	9.300	9.311	0.010
**24**	401.9	> 635	1.58	8.651	9.311	0.660
**Geometric Mean**	**155.8**	**409.1**	**2.63**	**7.284**	**8.676**	**1.392**
**(95% CI)**	**(107.0 – 227.1)**	**(317.7 – 526.9)**	**(2.56 – 2.69)**	**(6.741 – 7.827)**	**(8.311 – 9.041)**	**(1.357 – 1.428)**

Negative tests (PD_{15} > 635 mg) have a PD_{15} value of 635 mg imputed for use in calculations.
CI: confidence interval; DD: dose doubling; PD_{15}: provoking dose at 15% drop in FEV_1.

Practical Considerations for the conduct of the Mannitol Challenge Test

Source:

Adapted from Patient Information Leaflet (United Kingdom) Dated 12/2018

https://www.medicines.org.uk/emc/files/pil.4809.pdf (accessed 15 September 2021)

(Reproduced with permission from Pharmaxis Ltd.)

Contraindications

Known hypersensitivity to mannitol or to any of the capsule ingredients.

The inhaled mannitol challenge test should not be given to patients with severe airflow limitation (FEV_1 < 50% predicted or < 1.0 l) or conditions that may be compromised by induced bronchospasm or repeated blowing manoeuvres. These include aortic or cerebral aneurysm, uncontrolled hypertension, myocardial infarction or a cerebral vascular accident in the previous six months.

Special warnings and precautions for use

The mannitol challenge is to be administered by inhalation only. Inhaled mannitol causes bronchoconstriction. The mannitol inhalation test should only be conducted in suitable laboratories/clinics under the supervision of an experienced physician and by a physician or another health professional appropriately trained to perform bronchial provocation tests and to manage acute bronchospasm. The responsible physician, appropriately trained to treat acute bronchospasm, including appropriate use of resuscitation equipment, must be close enough to respond quickly to an emergency. A stethoscope, sphygmomanometer, and pulse oximeter should be available. Patients should not be left unattended during the procedure once the administration of mannitol has begun.

Medications to treat severe bronchospasm must be present in the testing area. They include adrenaline for subcutaneous injection, and salbutamol or other beta agonists in metered-dose inhalers. Oxygen must be available. A small-volume nebuliser should be readily available for the administration of bronchodilators.

General precautions when conducting spirometry and bronchial provocation testing should be observed, and caution should be exercised in patients with the following: ventilatory impairment (baseline FEV_1 of less than 70% of predicted normal values or an absolute value of 1.5 l or less in adults), spirometry induced bronchoconstriction, haemoptysis of unknown origin, pneumothorax, recent abdominal or thoracic surgery, recent intraocular surgery, unstable angina, inability to perform spirometry of acceptable quality or upper or lower respiratory tract infection in the previous 2 weeks.

If a patient has spirometry induced asthma or the FEV_1 fall following the 0 mg capsule is greater than 10%, a standard dose of bronchodilator should be given and the mannitol challenge discontinued.

The mannitol test should not be used in patients below 6 years of age due to their inability to provide reproducible spirometric measurements.

There is limited information on the use of inhaled mannitol as a challenge test in patients 6-18 years of age therefore mannitol is not recommended in this population.

The effects of repeat mannitol testing within a short period of time have not been investigated therefore careful consideration should be given to repeat use of mannitol.

Equipment

Mannitol Challenge Kit (containing mannitol capsules, inhaler device and instruction leaflet)

Spirometer & mouthpiece

Nose clip

Timer (which can be set to 60 seconds)

Calculator

Bronchodilator (e.g. salbutamol)

Oxygen and other relevant emergency equipment should be readily available as per standard Bronchial Provocation Testing protocols.

Recommended Medication Withholding Times

Failure to withhold medications may affect the results of the mannitol challenge.

Recommended periods for withholding medications are generally based on their duration of action.

**Time to Withhold**	**Medication**
6 – 8 hours	INHALED NON-STEROIDAL ANTI-INFLAMMATORY AGENTS ***e.g. sodium cromoglycate, nedocromil sodium***
8 hours	SHORT-ACTING BETA$_2$ AGONISTS ***e.g. salbutamol, terbutaline***
12 hours	INHALED CORTICOSTEROIDS ***e.g. beclomethasone, budesonide, fluticasone***
12 hours	IPRATROPIUM BROMIDE
24 hours	INHALED CORTICOSTEROIDS PLUS LONG-ACTING BETA$_2$ AGONISTS ***e.g. fluticasone and salmeterol, budesonide and formoterol***
24 hours	LONG-ACTING BETA$_2$ AGONISTS ***e.g. salmeterol, formoterol***

24 hours	THEOPHYLLINE
72 hours	TIOTROPIUM BROMIDE
72 hours	ANTIHISTAMINES ***e.g. cetirizine, fexofenadine, loratadine***
4 days	LEUKOTRIENE-RECEPTOR ANTAGONISTS ***e.g. montelukast***

Exercise: Vigorous exercise should be fully avoided on the day of the test, as this may affect test results.

Smoking: Since smoking may affect test results it is recommended that patients refrain from smoking for at least 6 hours prior to testing.

Food: Ingestion of significant quantities of coffee, tea, cola drinks, chocolate or other foods containing caffeine may decrease bronchial responsiveness and should be totally avoided on the day of the test.

Inhaler instructions

These instructions show you how to use the inhaler device.

1. **Remove Cap:** Using both hands, hold the inhaler upright and remove the cap.

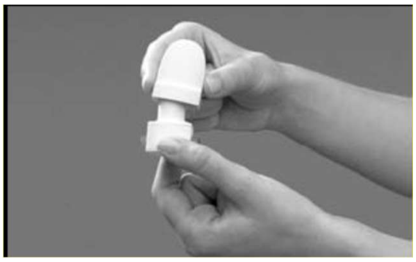

2. **Open:** Hold the base of the inhaler firmly with one hand and open the device by rotating the mouthpiece in the direction of the arrow as shown.

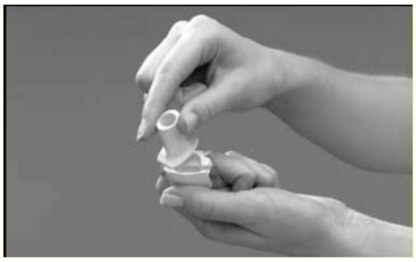

3. **Load:** Make sure your hands are dry, remove a capsule from the mannitol pack and place into the inhaler as illustrated.

It does not matter which way the capsule is placed in the chamber.

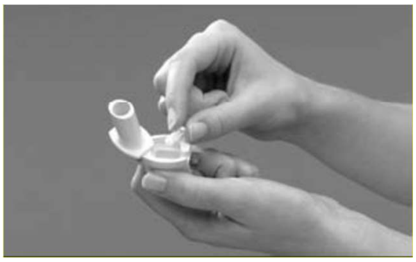

4. **Close:** Keeping the device in an upright position, twist the mouthpiece into the closed position until you hear it 'click'.

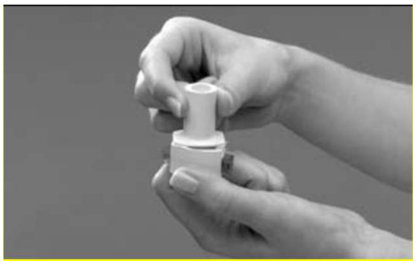

5**. Pierce Capsule:** Hold the inhaler upright and fully depress both piercing buttons on the sides of the device at the same time. Do this once only, since piercing the capsule more than once may cause it to split/fragment. The piercing action makes holes in the capsule and allows the powder in the capsule to be released during inhalation.

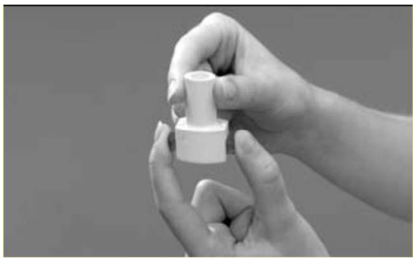

6. **Prepare for Inhalation:** Tilt the inhaler so that the mouthpiece faces slightly downward at a 45 degree angle as shown on the picture below, until the capsule drops forward into the spinning chamber. Keep the device tilted in this way and instruct the patient to breathe out completely (away from the inhaler).

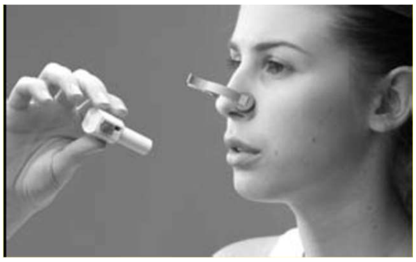

7. **Inhale:** The patient should tilt their head back slightly, and keeping the inhaler at a 45 degree angle, raise the device to their mouth and ensure they close their lips tightly around the mouthpiece. Encourage the patient to take a controlled rapid and deep inspiration to fill the lungs. The patient should then hold their breath for five seconds.

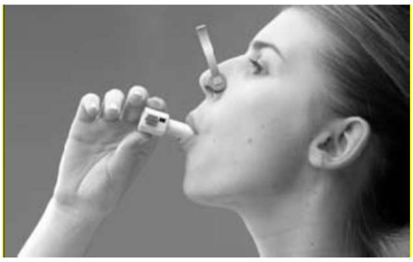

Note: During a successful inhalation you should hear a 'rattling' sound as the capsule spins in the inhaler.

8. **Exhale:** Remove the inhaler from the patient's mouth, allow them to exhale and resume normal breathing.

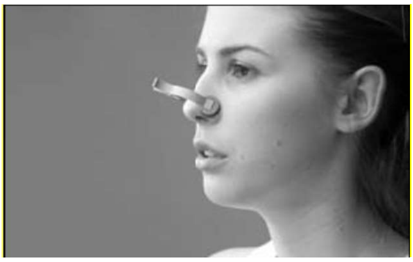

9. **Check:** The mannitol capsule must spin in the inhaler in order to empty.

A second inhalation (using the same capsule) may be required immediately if the capsule is not empty following inhalation. Check the capsule following each inhalation.

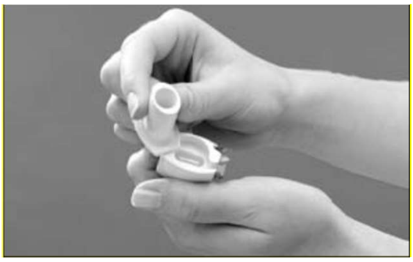

Please Note:

The inhaler device is designed for SINGLE USE ONLY (one device per challenge) and should not be cleaned during the challenge.

Discard the inhaler following each mannitol challenge. The inhaler must not be sterilised or re-used as this may compromise the integrity of subsequent test results.

Inhaler devise

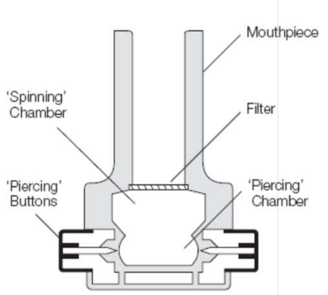

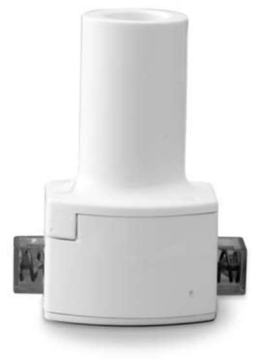

Procedure Guidelines

STEP 1: Make sure the patient has withheld the following medications.

STEP 2: The patient should be seated for the test. Explain the procedure; include what is required for an FVC manoeuvre and FEV_1 measurement and the type of inspiratory flow required for the inhaler. Demonstrate as required.

STEP 3: Enter the patient's details in the spirometer (age, height, race, date of birth, gender e.t.c.).

STEP 4: Determine the pre-challenge FEV_1. Ask the patient to perform an FVC manoeuvre according to the ATS/ERS guidelines, perform three acceptable manoeuvres of which two are reproduced. Use the highest value as pre-challenge FEV_1.The patient's FEV_1 should be ≥ 70% of the predicted value.

Caution should be used in patients with an FEV_1 of less than 70% of the predicted value.

STEP 5: Calculate the baseline FEV_1 (0 mg)

a. Remove the **0 mg mannitol** capsule from the blister, twist open the inhaler (as per the arrow on the device), place the capsule inside and close the device.

b. Pierce the capsule once only by depressing the coloured buttons on either side of the inhaler.

c. Ask the patient to put on the nose clip, and breathe through their mouth.

d. Tilt the inhaler at a 45° angle (mouthpiece down). Check the capsule has moved from the piercing chamber into the spinning chamber closest to the mouthpiece.

You can often hear the capsule fall forward or see the capsule through the vents on each side of the device. Give the inhaler to the patient ensuring that they keep the inhaler at the same angle.

e. Ensure the patient is sitting up straight. Ask the patient to exhale (away from the inhaler), seal their lips around the inhaler mouthpiece and take a controlled rapid and deep inspiration until their lungs are full. During successful inhalation you should hear a 'rattling' sound as the capsule spins within the device.

f. At the end of the patient's inhalation, start a 60 second timer, and ask the patient to hold their breath for 5 seconds. When 5 seconds has passed, instruct the patient to exhale through their mouth (away from the inhaler), remove the nose clip and breathe normally.

g. When the timer beeps after 60 seconds, immediately instruct the patient to perform two acceptable FEV_1 measurements. These measurements must be within 0.15 l (150 ml) variability. If there is greater than 0.15 l variability between readings instruct the patient to perform another FEV_1. **Record the highest FEV_1 reading as the baseline FEV_1**. *If the highest FEV_1 is ≥ 10% lower than the pre-challenge FEV_1 do not continue with the test.*

h. **Calculate the target FEV_1**

A positive mannitol challenge result is achieved when the patient's FEV_1 falls ≥ 15% from their baseline FEV_1. To calculate the target FEV_1, multiply the baseline FEV_1 (the highest reading obtained at 0 mg) obtained above by 0.85. Record this value.

STEP 6: 5mg capsule

a. Insert 5 mg capsule into the inhaler and pierce as in Step 5.

b. Repeat as in steps 5c – f above.

c. Following inhalation remove the capsule from the inhaler and check to ensure it has been emptied completely, if not, a 2nd inhalation will be required immediately.

d. Load the 10 mg capsule in readiness for the next dose.

e. At 60 seconds following inhalation, immediately measure the patient's FEV_1 twice *(acceptability criteria must be met)*. Use the highest of these two values to calculate the change in FEV_1.

f. Compare the FEV_1 value at this dose to the target FEV_1. If the FEV_1 value is equal to or below the target value, or there has been an incremental fall of ≥ 10% from the previous dose, the challenge is positive and complete. If not, immediately proceed to next dose step.

STEP 7: 10 mg, 20 mg, 40 mg capsules

Administer the 10 mg, 20 mg and 40 mg doses following the directions given above (in step 6) for the 5 mg dose.

STEP 8: 80 mg dose (2 x 40 mg capsules)

a. Insert and pierce the first of the 40 mg capsules that comprise the 80 mg dose.

b. The patient should inhale the dose in the same manner as previous doses, hold their breath for 5 seconds and exhale.

c. Remove the first 40 mg capsule from device and check to ensure it has been emptied completely, if not, a 2nd inhalation will be required immediately. Do this following the administration of every capsule.

d. Following inhalation, load the second 40mg capsule and offer to the patient immediately following exhalation.

e. Instruct the patient to inhale the 2nd capsule immediately to ensure that the osmotic effect of mannitol is cumulative.

f. Activate timer at the end of the 2nd capsule inhalation.

g. Instruct the patient to hold their breath for 5 seconds before exhaling.

h. At 60 seconds following inhalation of the 2nd capsule, immediately measure the patient's FEV_1 twice *(acceptability criteria must be met)*. Use the higher of these two values to calculate the change in FEV_1.

i. Compare the FEV_1 value at this dose to the target FEV_1. If the FEV_1 value is equal to or below the target value, or there has been an incremental fall of ≥ 10%, the challenge is positive and complete. If not, immediately proceed to next dose step.

<u>STEP 9:</u> 1st of 160 mg dose (4 x 40 mg capsules)

a. Insert and pierce the 1st of the 40 mg capsules that comprise the 160 mg dose.

b. The patient should inhale the dose in same manner as previous doses, hold their breath for 5 seconds and exhale.

c. Remove capsule from device and check to ensure it has been emptied completely, if not, a 2nd inhalation will be required immediately. Do this following the administration of every capsule.

d. Following inhalation, load the 2nd 40 mg capsule and offer to the patient immediately following exhalation.

e. The patient should inhale contents of the 2nd capsule, hold their breath for 5 seconds and exhale.

f. Following inhalation, load the 3rd 40 mg capsule and offer to the patient immediately following exhalation.

g. The patient should inhale the contents of the 3rd capsule, hold their breath for 5 seconds and exhale.

h. Immediately following inhalation, load the 4th 40 mg capsule and offer to the patient immediately following exhalation.

i. Instruct the patient to inhale the 4th capsule immediately to ensure that the osmotic effect of mannitol is cumulative.

j. Activate timer at the end of the 4th capsule inhalation.

k. Instruct the patient to hold their breath for 5 seconds, before exhaling.

l. At 60 seconds following inhalation of the 4th capsule, immediately measure the patient's FEV_1 twice *(acceptability criteria must be met)*. Use the higher of these two values to calculate the change in FEV_1.

m. Compare the FEV_1 value at this dose to the target FEV_1. If the FEV_1 value is equal to or below the target value, or there has been an incremental fall of ≥ 10% from the previous dose the challenge is positive and complete. If not, immediately proceed to next dose step.

STEP 10: 2nd x 160 mg dose (4 x 40 mg capsules)

Administer the 2nd 160 mg dose following the directions given above in step 9.

STEP 11: 3rd x 160 mg dose (4 x 40 mg capsules)

Administer the 3rd 160 mg dose following the directions given above in step 9.

At the completion of this dose, 635 mg has been administered. Providing a positive response has not been met, the challenge should be considered negative and complete.

STEP 12: Following completion of the challenge with a positive result you should administer a bronchodilator and monitor the patient for 15 minutes to ensure their FEV_1 has returned to within 5% of pre-challenge level. (In the case of a negative result you may or may not wish to give a bronchodilator).

Mannitol Test Worksheet

ID-label:

Performed by: _______
Date: _______________

- Check contraindications and medication to withhold
- Measure FEV_1 and calculate FEV_1 % predicted
- Calculate cut-off FEV_1 for a positive test (0.85 x FEV_1 after 0 mg capsule)
- Instruct patient to hold breath for 5 seconds after inhalation of each capsule
- Measure FEV_1 x 2, 60 seconds after the last capsule of each dose is inhaled. The highest value is registered
- Maximum duration of test: 35 minutes

FEV_1-start: ___________L = ___________% (Only perform test if $FEV_1 \geq 70\%$ of predicted)

Test commenced (time): __________ Test completed (time): __________ (max 35 minutes)

Calculate cut-off for positive test: 15% fall = 0.85 x FEV_1 after 0 mg capsule

		Test is positive if FEV_1 falls below: __________ L
Dose	Cummulated dose (mg)	FEV_1 (L)
0	0	
5	5	
10	15	
20	35	
40	75	
80 (2x40)	155	
160(4x40)	315	
160(4x40)	475	
160(4x40)	635	

Give standard inhalation of SABA after last dose

Medications to withhold prior to the test

SABA: 8 hours
SAMA: 12 hours
LABA: 24 hours
ICS: 12 hours
ICS+LABA: 24 hours
LAMA: 72 hours

Leukotriene-receptor antagonists: 4 days

References

1. Barben J, Roberts M, Chew N, Carlin JB, Robertson CF. Repeatability of bronchial responsiveness to mannitol dry powder in children with asthma. Pediatr Pulmonol. 2003;36(6):490-4.
2. Udesen PB, Westergaard CG, Porsbjerg C, Backer V. Stability of FeNO and airway hyperresponsiveness to mannitol in untreated asthmatics. J Asthma. 2017;54(5):530-6.
3. Brannan JD, Anderson SD, Freed R, Leuppi JD, Koskela H, Chan HK. Nedocromil sodium inhibits responsiveness to inhaled mannitol in asthmatic subjects. Am J Respir Crit Care Med. 2000;161(6):2096-9.
4. Brannan JD, Anderson SD, Gomes K, King GG, Chan HK, Seale JP. Fexofenadine decreases sensitivity to and montelukast improves recovery from inhaled mannitol. Am J Respir Crit Care Med. 2001;163(6):1420-5.
5. Anderson WJ, Short PM, Williamson PA, Lipworth BJ. Inhaled corticosteroid dose response using domiciliary exhaled nitric oxide in persistent asthma: the FENOtype trial. Chest. 2012;142(6):1553-1561.
6. Clearie KL, McKinlay L, Williamson PA, Lipworth BJ. Fluticasone/Salmeterol combination confers benefits in people with asthma who smoke. Chest. 2012;141(2):330-8.
7. Brannan JD, Bood J, Alkhabaz A, Balgoma D, Otis J, Delin I, et al. The effect of omega-3 fatty acids on bronchial hyperresponsiveness, sputum eosinophilia, and mast cell mediators in asthma. Chest. 2015;147(2):397-405.
8. Baraket M, Oliver BG, Burgess JK, Lim S, King GG, Black JL. Is low dose inhaled corticosteroid therapy as effective for inflammation and remodeling in asthma? A randomized, parallel group study. Respir Res. 2012;13(1):11.
9. Toennesen LL, Meteran H, Hostrup M, Wium Geiker NR, Jensen CB, Porsbjerg C, et al. Effects of Exercise and Diet in Nonobese Asthma Patients-A Randomized Controlled Trial. J Allergy Clin Immunol Pract. 2018;6(3):803-11.
10. Diver S, Khalfaoui L, Emson C, Wenzel SE, Menzies-Gow A, Wechsler ME, Johnston J, Molfino N, Parnes JR, Megally A, Colice G, Brightling CE. Effect of tezepelumab on airway inflammatory cells, remodelling, and hyperresponsiveness in patients with moderate-to-severe uncontrolled asthma (CASCADE): a double-blind, randomised, placebo-controlled, phase 2 trial. Lancet Respir Med. 2021 Jul 9:S2213-2600(21)00226-5. Epub ahead of print.
11. Asger Sverrild , Susanne Hansen, Morten Hvidtfeldt, Carl-Magnus Clausson, Olga Cozzolino, Samuel Cerps, Lena Uller, Vibeke Backer, Jonas Erjefält, Celeste Porsbjerg.The effect of tezepelumab on airway hyperresponsiveness to mannitol in asthma (UPSTREAM) Eur Respir J.; 2021 Epub ahead of print.

12. Brannan JD, Koskela H, Anderson SD, Chan HK. Budesonide reduces sensitivity and reactivity to inhaled mannitol in asthmatic subjects. Respirology. 2002;7(1):37-44.

13. Koskela HO, Hyvärinen L, Brannan JD, Chan HK, Anderson SD. Sensitivity and validity of three bronchial provocation tests to demonstrate the effect of inhaled corticosteroids in asthma. Chest. 2003;124(4):1341-9.

14. Kersten ET, Driessen JM, Duiverman EJ, Thio BJ. The effect of stepping down combination therapy on airway hyperresponsiveness to mannitol. Respir Med. 2011;105(5):691-7.

15. Currie GP, Haggart K, Brannan JD, Lee DK, Anderson SD, Lipworth BJ. Relationship between airway hyperresponsiveness to mannitol and adenosine monophosphate. Allergy. 2003;58(8):762-6.

16. McClean MA, Htun C, King GG, Berend N, Salome CM. Cut-points for response to mannitol challenges using the forced oscillation technique. Respir Med. 2011 Apr;105(4):533-40.

17. Currie GP, Haggart K, Lee DK, Fowler SJ, Wilson AM, Brannan JD, et al. Effects of mediator antagonism on mannitol and adenosine monophosphate challenges. Clin Exp Allergy. 2003;33(6):783-8.

18. Koskela HO, Martens R, Brannan JD, Anderson SD, Leuppi J, Chan HK. Dissociation in the effect of nedocromil on mannitol-induced cough or bronchoconstriction in asthmatic subjects. Respirology. 2005;10(4):442-8.

19. Jabbal S, Manoharan A, Lipworth BJ. Bronchoprotective tolerance with indacaterol is not modified by concomitant tiotropium in persistent asthma. Clin Exp Allergy. 2017;47(10):1239-45.

20. Lussana F, Di Marco F, Terraneo S, Parati M, Razzari C, Scavone M, et al. Effect of prasugrel in patients with asthma: results of PRINA, a randomized, double-blind, placebo-controlled, cross-over study. J Thromb Haemost. 2015;13(1):136-41.

21. Lipworth B, RuiWen Kuo C, Jabbal S, Chan R. Inhaled triple therapy and airway hyperresponsiveness in persistent asthma. Ann Allergy Asthma Immunol. 2021 May;126(5):597-598. Epub ahead of print.

22. Török S, Mueller T, Miedinger D, Jochmann A, Zellweger LJ, Sauter S, et al. An open-label study examining the effect of pharmacological treatment on mannitol- and exercise-induced airway hyperresponsiveness in asthmatic children and adolescents with exercise-induced bronchoconstriction. BMC Pediatr. 2014;14:196.
